# M2e-based universal influenza vaccines: a historical overview and new approaches to development

**DOI:** 10.1186/s12929-019-0572-3

**Published:** 2019-10-19

**Authors:** Daria Mezhenskaya, Irina Isakova-Sivak, Larisa Rudenko

**Affiliations:** Department of Virology, Institute of Experimental Medicine, 12 Acad. Pavlov Street, St. Petersburg, 197376 Russia

**Keywords:** Influenza a virus, Influenza M2 ectodomain, Conserved protein, Cross-protection, Universal influenza vaccine

## Abstract

The influenza A virus was isolated for the first time in 1931, and the first attempts to develop a vaccine against the virus began soon afterwards. In addition to causing seasonal epidemics, influenza viruses can cause pandemics at random intervals, which are very hard to predict. Vaccination is the most effective way of preventing the spread of influenza infection. However, seasonal vaccination is ineffective against pandemic influenza viruses because of antigenic differences, and it takes approximately six months from isolation of a new virus to develop an effective vaccine. One of the possible ways to fight the emergence of pandemics may be by using a new type of vaccine, with a long and broad spectrum of action. The extracellular domain of the M2 protein (M2e) of influenza A virus is a conservative region, and an attractive target for a universal influenza vaccine. This review gives a historical overview of the study of M2 protein, and summarizes the latest developments in the preparation of M2e-based universal influenza vaccines.

## Introduction

The extremely high variability in the antigenic properties of influenza virus is related to the structure of its genome that allows reassortment and the structural flexibility of the viral glycoproteins, which tolerate amino acid residue substitutions in the major antigenic sites without loss of function. Often immunity raised against previously circulating variants is not capable of protecting against newly emerging drift variants. In addition, completely new antigenic variants of the influenza virus are occasionally introduced into the human population; since the population is immunologically naive to them, these viruses spread easily and can cause pandemics. These features of influenza infection explain why there is interest around the world in developing a universal influenza vaccine, which could induce a cross-reactive immune response to the most conservative parts of the viral proteins.

Classic influenza vaccines commonly induce antibodies to the viral surface antigens, hemagglutinin (HA) and neuraminidase (NA), mainly to their immunodominant hypervariable regions. Constant antigenic drift allows the virus easily to escape the action of these antibodies, reducing the effectiveness of vaccination and leading to a need for annual updating of vaccine strains in the seasonal influenza vaccines [95, 129]. The performance of seasonal influenza vaccines can be improved by increasing the speed of vaccine production and using new adjuvants and new vaccination strategies. However, these vaccines will not be able to protect against newly emerging pandemic influenza viruses because of significant antigenic differences. Potentially pandemic viruses have been identified and many vaccines, based on different approaches and platforms, have been developed against them [133, 134]. However, these vaccines also have narrow specificity and may not have cross-reactivity, even within a single subtype. There has, therefore, been significant interest in the development of new vaccines that would have a longer and wider spectrum of action [9], and which would provide long-term protection not only against drifted variants of influenza A viruses of one subtype but also against viruses of other subtypes. One approach to expanding the spectrum of the protective action of influenza vaccines is to enhance the induction of cross-reacting immune response factors that target highly conserved antigens in influenza viruses of various subtypes [61, 62]. The influenza virion contains multiple conservative domains that, because of their functional significance, are rather weak immunogens; classical approaches to immunization are not able to induce a strong immune response to these sites.

The broadly protective vaccines currently being developed can be divided into two groups.

1. Vaccines that induce antibodies to structurally conserved regions of viral proteins, such as the stalk domain of the hemagglutinin which is important for the penetration of the influenza virus into the cell [63]; broadly immunogenic epitopes located at the contact surface between HA head domains [5, 130]; the enzymatic site of NA, where the surface cell sialic acids are cut [23]; and the ectodomain of the M2 protein (reviewed in this manuscript). Induced antibodies can both have a neutralizing function and participate in the process of antibody-dependent cellular cytotoxicity (ADCC) or phagocytosis (ADCP), thereby accelerating the elimination of the virus from an infected organism [126].
2. Vaccines that induce a cross-reactive T-cell immune response to the conserved epitopes of the virion’s internal proteins, such as nucleoprotein (NP) and M1 [27]. This is supported by a strong correlation of CD8+ T cells specific to conserved viral epitopes with cross-protection against symptomatic influenza in the absence of cross-reactive neutralizing antibodies [111].

## Historical aspects of studying M2 protein

Most attempts to create a universal influenza vaccine have been based on the M2e epitopes. The first evidence of the existence of M2 protein was published in 1981 [65]. Several groups had already shown that the influenza A virus has a segmented genome consisting of 8 elements [84, 90, 98]. It was also known that the 8th mRNA encodes two proteins, NS1 and NS2 [66], and that the nucleotide sequence of the 7th mRNA contains two open reading frames [135], which were thought to encode the already discovered M1 protein and a previously unknown protein consisting of 97 amino acid residues [67].

Further study using viral lysate labelling with [^35^S] methionine and subsequent separation in a polyacrylamide gel detected a new protein with greater electrophoretic mobility than the already known influenza A protein. Isolation of different mRNA segments followed by peptide synthesis in vitro produced a new peptide of similar molecular weight; it was concluded that the 7th segment of the mRNA of the influenza A genome and the new M2 protein were genetically related. RNA-RNA hybridization established that the 7th segment encodes M1 and M2 proteins [65].

At first, M2 was detected only in infected cells [65], and not in the influenza virions themselves, which made it possible to put forward an assumption about the specific location of this protein. On the basis of the amino acid sequence of M2 [67], a hydropathy graph was constructed indicating the presence of a hydrophobic domain. Further study of the protein led to the conclusion that the C-terminus is located in the cytoplasmic space, while the N-terminal part of M2 is exposed at the viral surface [69].

The first monoclonal antibody to M2 (14C2) was obtained in 1988 as a result of the immunization of BALB/c mice with purified M2 protein along with Freund’s adjuvant. A subsequent study of 14C2 showed that the antibody binds to the extracellular N-terminal region of M2 (M2e) [140]. Currently, there is evidence that the binding site is a fragment of M2e from the 6th to the 15th residue [128]. In 1998, it was shown that the 14C2 antibody is not able to inhibit the adsorption and penetration of influenza virus into cells, but is able to limit the growth of influenza A virus in vitro, though this restriction is highly strain dependent [140]. Another M2e-specific monoclonal antibody was obtained in 1996. To this end, splenocytes from BALB/c mice immunized with the keyhole limpet hemocyanin-linked M2 peptide SLLTEVETPIRNEWGCRCND were fused with F23.1 hybridoma cells producing an immunoglobulin G2a (IgG2a) monoclonal antibody. The authors selected a monoclonal antibody (3F12), which not only had cross-reactivity to different influenza A strains, but could also bind to the T-cell receptor. It was also shown that such a bispecific antibody is able to limit the growth of influenza A virus in vitro (with less efficiency than 14C2) and redirect activated T-cells to eliminate infected cells [30].

In 2003, another study was published in which rabbits were immunized with 4 peptides from the N-terminal section of M2, with overlapping areas: а.a. 2–24, а.a. 2–12, а.a. 8–18 and а.a. 13–24. Further study showed that they all produced antibodies capable of recognizing each of the four peptides. It has also been shown that antibodies recognizing the N-terminus of M2 (a.a 2–12) have virus-neutralizing activity against both influenza A and B in vitro [77]. The authors suggested that this part of M2 may contain one epitope that can induce antibodies with inhibitory activities against both viruses, but there is no additional evidence that could confirm that anti-M2e immunity can suppress influenza B virus replication. Later, the same group of scientists reported that a small area of M2 EVETPIRN (a.a. 6–13) can produce antibodies 8C6 and 1B12, but their protective effect has not yet been sufficiently studied [78].

One of the potential therapeutic antibodies is TCN-032, which has passed phase I and II clinical trials. TCN-032 recognizes the M2e2–6 epitope [37] and may provide immediate immunity against influenza A infection [99]. Another potential therapeutic antibody is anti-M2e antibody Z3G1, which has been shown to protect mice applied therapeutically at different time points after infection [128].

Mutational analysis, crystallography analysis of the structure and nuclear magnetic resonance have established that M2 is a transmembrane protein, consisting of four α-helices. Each helix has two conservative residues (His37 and Trp41), which play an important role in the life cycle of influenza virus [115]. Highly conserved and oxidized cysteine residues at positions 17 and 19 are responsible for the stabilization of the structure [44].

The entire M2 protein can be divided into 3 parts: the N-terminal or extracellular domain (23 a.a., excluding the 1st methionine); the hydrophobic transmembrane domain (19 a.a.); and the C-terminal domain (54 a.a.). The extracellular domain of M2 (M2e) consists of 23 amino acid residues and is a highly conserved region in all influenza A viruses. One of the reasons for the low variability of M2e is its inability to induce an immune response in an infected organism [11], so there is no selection pressure on the site. A more important reason is believed to be the genetic relationship between M2e and M1: a.a. 1–9 of M2e and M1 are encoded by the same nucleotides in the same reading frame. Amino acids 10–23 of M2e and a.a. 239–252 of M1 are also encoded by the same RNA sequence but are translated in different reading frames [51]. M1, in turn, is a highly conserved matrix protein with a countable number of currently known mutations [31].

**Fig. 1:**
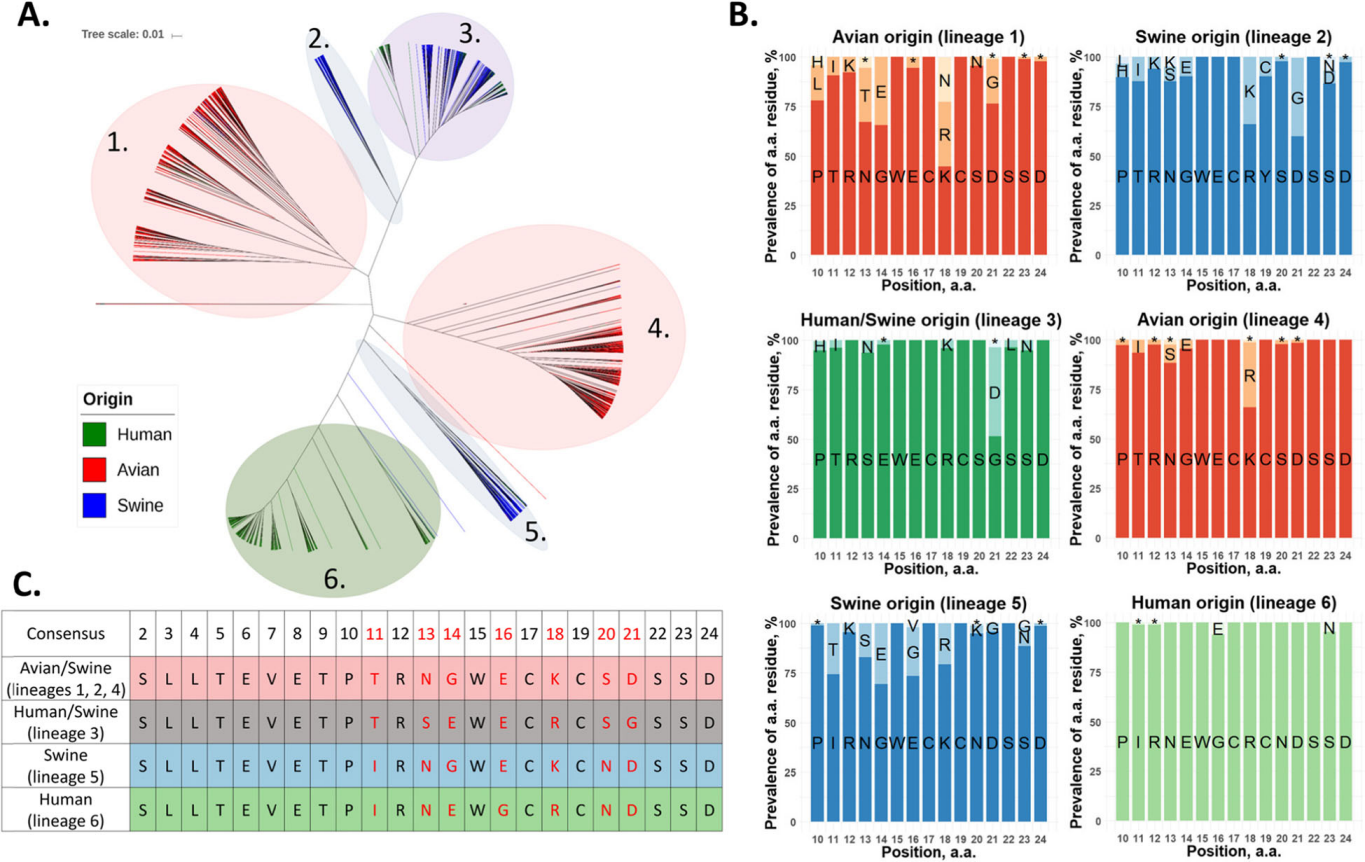
Analysis of M2e sequences of human, swine and avian influenza viruses. **a** Phylogenetic tree was generated from 27,253 human, 15,367 avian and 5379 swine individual sequences of influenza A virus M gene (obtained from the Influenza Research Database (fludb.org)) using MAFFT. The tree was visualized using the Interactive Tree Of Life (iTOL) server. **b** Prevalence of amino acid residues in M2e protein of selected M2e lineages. The consensus sequences for each lineage were obtained using UGENE Multiple Alignment software. Residues with a frequency below 3% are indicated with (*). **c** Proposed consensus M2e sequences for the development of a universal influenza vaccine

The whole of M2 is highly conserved, but its extracellular part is of the greatest interest as a potential antigen. We analyzed 27,253 human, 15,367 avian and 5379 swine individual sequences of influenza A virus M gene (obtained from the Influenza Research Database (fludb.org)) and generated a phylogenetic tree using MAFFT [55]. The tree was visualized using the Interactive Tree Of Life (iTOL) server [76]. As shown on Fig. 1a, the M2e sequences of influenza A viruses have evolutionary diverged into several lineages mainly related to host species. The generated phylogenetic tree basically repeats the published analysis of Furuse et al. [33], but in contrast to this study published before the 2009 pandemic, our analysis included a large number of sequences of H1N1pdm09 virus M gene, which is known to originate from the Eurasian avian-like swine H1N1 lineage [109]. We selected six different lineages of M2e protein based on their phylogenetic relatedness and obtained consensus sequences for each lineage using UGENE Multiple Alignment software (Fig. 1b). As mentioned above, the first nine amino acids have the lowest variability for each analyzed group. The remaining residues have different percentage mutations for each origin; however, the residues Arg12, Trp15, Cys17, Cys19, Ser22 and Asp24 have the lowest frequency of mutations, which may reflect their functional significance. The only exception is the residue at 19th position in the swine influenza viruses that fall into lineage 2, in which Tyr dominated in this position (Fig. 1b). The slight difference between the consensus sequences is also significant for the choice of the appropriate sequence for a universal vaccine. For example, immunization of pigs with human M2e using different carriers did not protect the animals from the lethal swine subtype of influenza A virus [41]. It has also been shown that monoclonal antibody 8C6, which protects mice against lethal challenge virus [78], is able to recognize the consensus part of human M2e “EVETPIRN” sequence (а.a. 6–13), but not the consensus fragment of avian M2e “EVETPTRN” (a.a. 6–13). Thus, it is possible that an amino acid substitution at the 11th position in avian M2e may allow avian influenza A virus to escape the immune response in humans [79]. Based on our analysis of all available M2e sequences, in order to develop a M2e-based universal influenza vaccine we propose using four different consensus M2e protein sequences that will cover most human, avian and swine influenza A viruses (Fig. 1c). In contrast to previously published studies, we suggest using two different M2e sequences for human influenza viruses due to the significant variations between the human viruses isolated before the 2009 pandemic and the H1N1pdm09 viruses.

The structure of free M2e is currently unknown. However, a number of studies have obtained crystallographic data on the structures of “M2e + antibody”. Using the example of MAb65, MAb148, Fab148 and Fab65 antibodies, it has been shown that M2e can take at least two different conformations to increase its affinity. For example, interaction with Fab148 and MAb148 occurs through residues 2–5, which play an important role in the interaction and form a β-turn. For interaction with MAb65, not only do residues 5–8 form a β-turn, but also the whole M2e takes a U-shaped formation with a central Trp15 [15]. For the formation of a stable complex with the Fab65 antibody, the importance of residues 10–11 in M2e has been shown, while residues 6, 8 and 14 are responsible for hydrophilic interactions with MAb65 [16].

Study of the structural features of the transmembrane domain of M2 (a.a. 18–60) and nearby areas at pH 7.5 revealed the presence of a nonstructural N-terminal region (a.a. 18–23), a channel-forming transmembrane helix (a.a. 25–46), a short flexible loop (a.a. 47–50) and a C-terminal aliphatic helix (a.a. 51–59) [104].

The 7th segment of the influenza A virus genome can be subjected to alternative splicing with the formation of M2 mRNA and mRNA3; the formation of M4 mRNA is also possible in some strains [100]. This process can proceed independently of other influenza A virus proteins [68] using only the host cell machinery. However, a number of papers have reported the control of M1 mRNA splicing using other influenza A virus proteins [10, 100, 106, 107]. Currently, it is known that NS1 provides the main regulatory function of the amount of M2 in infected cells [10, 100], rather than polymerase proteins, as was previously thought [106, 107].

## Functions of M2 protein

Currently, several biological functions of M2 protein are known. The first and most important is as a proton-selective channel or so-called viroporin [97], which is formed by four fragments of H37xxxW41 [4, 118, 125]. Shortly after the influenza virus enters a cell as part of the endosome [42, 75, 132], M2 goes into an activated state triggered by the acidic environment inside the endosomes. Using the M2 protein, H^+^ ions are transferred through the viral membrane into the virion (proton transfer occurs through the formation of the protonated state of His37 [35, 125] and rotation of the indole of Trp41 [118]). The influx of protons also leads to an influx of potassium and sodium ions (K^+^ and Na^+^), which in turn lead to a change in the M1 conformation, and the viral RNA goes into a relaxed state [75, 112]. The low endosomal pH leads to conformational changes in the HA molecule, which in turn triggers the fusion of the virion membrane with the endosomal membrane. Further, the interaction between M1 and the ribonucleoprotein complex is weakened, which leads to the release of viral RNA into the cytosol [14]. It has also been shown that the ionic imbalance in the cell, resulting from the operation of the M2 protein, is a signal for the formation of inflammatory activity in myeloid cells such as macrophages and dendritic cells [49].

Another function of M2 is participation in the formation and budding of new influenza A virions. The study of this feature began with the discovery that viral replication is inhibited by antibodies that recognize the extracellular domain of M2, despite the small amount of M2 on the surface of influenza A virus [83]. Using reverse genetics and mutational analysis, it has been shown that truncated forms of M2, or forms with mutations in the cytoplasmic tail, further reduce the level of free M1 in the cellular environment, which is required for the assembly and budding of new virions [12]. It has also been shown that M2 protein mediates membrane budding and scission processes via a pathway that is independent of ESCRT (endosomal sorting complexes required for transport), unlike many other enveloped viruses [101]. The major role in M2-mediated budding and scission processes is played by the amphipathic region (residues 45–62), which interacts with various host cell proteins involved in remodeling the cell membrane (reviewed in [81]).

Furthermore, the M2 of influenza A virus is a mediator of macroautophagy inhibition at the stage of fusion of autophagosomes and lysosomes, which causes a subsequent increase in cell death as a result of influenza infection and an increase in viral antigen yield [7, 34].

The functional ortholog of influenza A virus M2 is M2 from influenza B virus (BM2), which also carries electrons through the membrane [86], interacts with the influenza B matrix 1 protein [127], crucial for virus assembly [50], and has an HxxxW motif in its transmembrane part [93]. However, the sequences of M2 and BM2 have significant differences. For example, the extracellular part of BM2 consists of only seven amino acid residues [102] (the consensus for human BM2e according to analysis of 9011 sequences from the Influenza Research Database is “MLEPFQI”). Because of its small size, BM2e is most likely not able to establish a protective immune responses [102].

A functional analogue of M2 is the M42 protein, which is obtained by splicing the 7th segment of the influenza A virus genome in some influenza strains [136].

Despite the importance of the M2 functions, it is not an essential protein for influenza A virus. In laboratory practice, so-called “M2 null” viruses have been obtained by various modifications of stop codons [13, 48, 131]. All of these viruses were viable but had a reduced replication level [13, 48, 116, 131, 136, 137]. Moreover, an M2-deficient single replication (M2SR) virus has been generated and evaluated as a possible candidate for live influenza vaccine. The M2SR vaccine was able to infect cells and express all viral proteins except M2, without generating progeny virus [103]. This vaccine platform proved efficient in protecting animals (mice and ferrets) against heterologous/heterosubtypic influenza virus infections [39, 40], and is now undergoing phase I clinical trials (clinicaltrials.gov NCT03553940; NCT02822105).

## Protection mechanisms of M2e-based vaccines

Despite the small size of M2e, many questions about this protein remain unanswered. For example, the defense mechanism of M2e-containing vaccines has not been fully clarified [71]. Since anti-M2e antibodies were discovered [140], it has been believed that they are the main mechanism of the protective action that has been repeatedly shown in various animal models [19, 22, 59]. Importantly, several groups have noted that M2e-based influenza vaccines induced a long-lasting M2e-specific antibody response [56, 105, 110]. The presence of antibody 14C2 reduces the expression level of viral protein M2 [45], thereby indirectly affecting the formation of new viral particles. However, it has been shown that influenza viruses that have mutations in the C-terminal region of M2 and the N-terminal region of M1 (even one amino acid substitution could be sufficient, for example, Pro10His, Val31Ile or Ala41Val) are resistant to the action of antibody 14C2 [139, 142].

Although the anti-M2e antibody are not neutralizing [28, 32, 89], their significant protective effect was demonstrated in multiple experiments, including studies on passive transfer of M2e immune serum or anti-M2e monoclonal antibodies [28, 60, 88, 121]. In addition, Eliasson et al. [26] showed that B cell-deficient mice are very poorly protected by a mucosal M2e-based vaccine even though these mice mount a considerable CD4 T cell response against M2e. In 2011, El Bakkouri et al. [24] demonstrated the crucial role of Fc gamma receptors (FcγR) in the in vivo protection afforded by M2e-specific IgG isotypes. In this study, wild-type and FcR γ^−/−^ BALB/c mice were passively immunized with anti-M2e immune serum, followed by lethal challenge with a mouse-adapted virus. Despite similar distribution of anti-M2e IgG titers and antibody isotype in both mouse strains, the FcR γ^−/−^ mice were significantly less protected than wild-type animals. Further experiments demonstrated that the activating receptor FcγRIII associated with the common γ-chain is required for anti-M2e IgG1 isotype-mediated in vivo immune protection [24]. Similar results were yielded in study by Lee et al. [72]: although the wild-type mice and FcR γ^−/−^ genotype mice had similar levels of antibodies (IgG1 and IgG2a) after immunization with M2e5x virus-like particles (VLP), the vaccine was significantly less protective in mice without an FcR γ-chain than in wild-type mice. Van den Hoecke et al. [124] showed that protection against influenza virus with different IgG antibody subclasses requires different FcR subtypes: protection with IgG1 requires FcγRIII, while IgG2a requires all three activating FcγRs.

It is known that M2 protein is expressed abundantly on the surface of infected cells, whereas only a few molecules are incorporated into the virion [140], therefore anti-M2e IgG antibody provide protection by interacting with virus-infected cells and triggering immune effector cell activation through its Fc region, resulting in killing and/or phagocytosis of the infected cells [24]. Many studies attempted to identify the effector cells which are responsible for elimination of M2-expressing cells in the presence of anti-M2e IgGs. Controversial results were yielded with respect to the formation of natural killer (NK)-mediated ADCC in response to immunization with M2e-containing vaccines. A number of studies have shown that the ADCC mechanism has an essential role of [53, 108, 128], whereas experiments with peptide-specific monoclonal antibodies have refuted this [32]. These studies [32, 53, 128] looked at the effect of NK-mediated ADCC during passive immunization, which introduces additional difficulties in identifying the true mechanism of protection. Using a conditional cell depletion protocol, El Bakkouri et al. [24] demonstrated that alveolar macrophages (AM) play a critical role in the protection mediated by anti-M2e IgG antibody. These results are of particular interest because AM are resident in the lung and are considered one the first immune cells to interfere with respiratory pathogens in the airways. The contribution of these components of the immune system alone is not sufficient for a complete defense, but it can influence innate immunity and the first stages of the adaptive immune response [110]. The activation of the complement system as a result of viral elimination after immunization with M2e-containing vaccines also raises questions [71]. The complement system can bind to influenza virions in the presence of virus-specific antibodies [8, 43]. A study by Wang et al. [128] showed the importance of the C3 complement system in reducing viral titer in the lungs of mice after challenge, although Jegerlehner et al. [53] found that the system does not play a significant role in protection.

Various studies have also demonstrated the importance of the M2e-specific CD4+ and CD8+ responses [25, 26, 57, 72, 119, 138]. CD8 T cells are known to kill target cells via perforin and FasL-mediated cytotoxicity pathways, thereby providing viral clearance [120], while CD4 cells influence the production of IFN-γ, which is involved in reducing viral titer [6]. Experiments on T-cell depletion showed the joint importance of CD4+ and CD8+ T cells in heterosubtypic cross-protection [57, 119], however different studies yielded discrepant results in M2e-specific T-cell responses, most probably due to the differences in immunization protocols, vaccine platforms and adjuvants, strains of animals, and routes of immunization [71]. A study by Eliasson et al. [26] demonstrated a critical role of M2e-specific lung-resident memory CD4, but not CD8, T cells induced by mucosal CTA1-3M2e-DD vaccine in protecting mice against a lethal influenza virus infection. Importantly, these CD4 T-cell responses were long-lasting, protecting animals even 12 months after vaccination [26]. In the study by Lee et al., the M2e5x VLP-immunized FcR γ^−/−^ mice recovered more quickly after viral exposure than naïve FcR γ^−/−^ mice, most probably due to the prevalence of IFN-γ-producing CD4 and CD8 T cells in the lungs of immunized FcR γ^−/−^ mice [72].

In summary, no complete defense mechanism valid for all M2e-containing vaccines has been found so far. All of the contradictory data mentioned can be explained by unaccounted mechanisms in the formation of protection or by the additional influence of the M2e carrier (studies with contradictory results used different types of carriers). Another factor may be the lack of a unified methodology. However, all researchers agree that the response to M2e-containing vaccines involves many key parts of the immune system, which can have a significant protective effect.

## Possible modifications of M2e

Most studies aimed at preparing a universal M2e-based influenza vaccine have used the whole M2 ectodomain (a.a. 2–24). However, truncated forms of M2e (Table 1) can be used to induce an immune response to important areas of M2e, reduce the cost of vaccine production and allow the use of new vectors that have a small capacity. For example, there are a number of antibodies (14C2, 3F12, 8C6, 1B12, Z3G1) [30, 78, 140] that recognize only a fraction of the total M2e, and that find wide application in practice.

**Table 1:** Antigenic variants of M2e fragments

Major feature	2	3	4	5	6	7	8	9	10	11	12	13	14	15	16	17	18	19	20	21	22	23	24	Reference
The most conservative part of M2e	S	L	L	T	E	V	E	T	P															[18, 51]
TCN-031 and TCN-032 antibodies recognition site	S	L	L	T	E																			[37]
O19 antibody recognition site	S	L	L	T	E	V	E	T																[32]
L18 antibody recognition site			L	T	E	V	E	T	P	I	R	N												
S1 antibody recognition site						V	E	T	P	I	R	N												
Influenza A and B virus neutralizing activity in vitro	S	L	L	T	E	V	E	T	P	I	R													[77]
Presence of B-cell epitope and 14C2 antibody recognition site					E	V	E	T	P	I	R	N												[78]
High affinity of binding with HLA-A2; presence of T-cell epitope		L	L	T	E	V	E	T	P	I														[36]
Potential epitope							E	T	P	I	R													[94]
Presence of CTL-cell epitopes	S	L	L	T	E	V	E	T	P	I	R	N	E	W	G									
Some critical residues for T-helpers																C	R	C	N	D	S	S	D	
Recognition of HLA-B44-restricted CD8+ CTL line 124						V	E	T	P	I	R	N	E	W										[52]
MHC class II H-2d-restricted epitope							E	T	P	I	R	N	E	W	G	S	R							[26]
L66 antibody recognition site	S	L	L	T	E	V	E	T	P	I	R	N	E	W	G									[128]
N547 antibody recognition site		L	L	T	E	V	E	T	P	I	R	N	E	W	G									
Z3G1 antibody recognition site		L	L	T	E	V	E	T	P	I	R													
C40G1 antibody recognition site								T	P	I	R	N	E											
14C2 antibody recognition site					E	V	E	T	P	I	R	N	E	W										

Induction of all kinds of anti-M2e antibodies is required for maximum protection of M2e-containing vaccines. However, these antibodies have different protective characteristics, and an optimum universal influenza vaccine may use only some of the M2e sites that induce the most effective antibodies (Table 1).

Fu et al. [32] compared different anti-M2e antibodies, and showed that the protective properties of L18 antibody (which recognizes the M2e 4–13 site) were superior to those of O19 (which recognizes the M2e 2–9 site), while S1 (recognizes the M2e 7–13 site) did not protect mice against lethal influenza A virus challenge. Data from other groups indicate that the M2e 4–13 region contains a B-cell epitope and a sequence recognized by the 14C2 antibody (M2e 6–13) [79], as well as an additional potential epitope (M2e 8–12) [94].

It is worth noting that, when shortened forms of the ectodomain M2 are used, and with regard to various defense mechanisms, the carrier of M2e epitopes plays an important role in immunogenicity. For example, immunization with a shortened form of M2e (2–16 amino acid residues) along with Freund’s adjuvant did not lead to the formation of a high level of anti-M2e antibodies [94], unlike when phage f88 was used as a carrier of M2e antigen [20]. It was concluded that a shortened form of M2e will produce a high level of anti-M2e antibodies only with a support system capable of inducing a T-cell response. This study also revealed the presence in M2e (17–24 a.a.) of amino acids important for the formation of a T-cell response.

## Development of M2e-based vaccines

The inhibition of replication of some influenza A strains by 14C2 antibody [140] merited further study since the extracellular part of the M2 protein is very conservative compared with HA and NA, and the cross-protective effect can be directed precisely at M2e. Study of the protective properties of 14C2 in BALB/c mice showed that it significantly reduced the replication of influenza A (but not influenza B) virus in lung tissue. This prompted the suggestion that the higher immunity of adults who have previously had influenza A infection compared with children is provided by the presence of M2e antibodies [121]. However, subsequent studies have shown that M2e itself is a weak immunogen and that only about half the people infected with influenza A virus are able to produce anti-M2e antibodies [143]. There is no reliable evidence that such a low concentration of antibodies plays any protective role [11, 143]. It has also been suggested that the introduction of M2e epitopes into the influenza vaccine will have a priming effect on the production of antibodies before subsequent influenza A virus infection [143].

Despite numerous reports of the low immunogenicity of M2e because of its small size, small number of copies in the virion, and the possible shielding effect of larger surface proteins of influenza A virus [29, 47, 140], it has been shown that immunization with free synthetic M2e peptide together with an adjuvant can produce high titers of anti-M2e antibodies that protect mice from lethal influenza challenge [138]. However, most attempts to create a universal M2e-based influenza vaccine have used different carriers. One of the first studies used a recombinant baculovirus containing M2 from influenza virus A/Ann Arbor/6/60 (Bac-AM2), to infect the *Spodoptera frugipedra* (Sf9) cell line. 14C2 antibody detection revealed the presence of M2 on the surface of infected cells. This allowed the use of Sf9 lysates infected with Bac-AM2 as a source of antigen to study the antibody response to M2 in people previously infected with influenza A virus [11].

One of the first prototypes of a universal influenza vaccine used hepatitis B core protein combined with the M2e peptide (M2HBc) as a carrier. In this design, the natural position of the N-terminal region of M2, located in the extracellular space, was simulated. Immunization of BALB/c mice with M2HBc resulted in a high level of protection against the lethal dose of influenza A virus, and led to the formation of anti-M2e antibodies, which were shown to be effective in passive immunization experiments [88]. Later various liposomal carriers [1], tobacco mosaic virus surface protein [96], and rotavirus NSP4 [3] were used to induce anti-M2e antibodies. Another carrier used was the GCN4 protein, which is a eukaryotic transcriptional protein activator. The use of M2e-tGCN4 resulted in significant production of M2e-specific antibodies, which protected vaccinated mice from a lethal dose of mouse-adapted influenza virus [17].

The most effective and cost-effective method is the creation of recombinant virus-like particles (VLPs), on the surface of which M2e would be represented [19]. Because M2 is a homotetramer consisting of two subunits linked by a disulfide bond, held together by covalent interactions [88], it has been suggested that M2e could also be used as a tetramer in the creation of recombinant constructs. In this approach, M2e will form a compactly folded protein, thereby ensuring the correct geometry of the virus particles. In addition, there are confirmations of the creation of VLPs consisting of five M2e tandem repeats (M2e5x). The intramuscular administration of M2e5x protected mice from influenza A viruses of different serotypes [56, 58, 74].

There are data on the highly conservative nature of Cys17 and Cys19 residues, although, in many studies, these residues were replaced by Ser17 and Ser19, respectively, in order to avoid protein aggregation due to the formation of disulfide bonds between M2e sites. Such substitutions are widely used because it has been shown that Cys17 and Cys19 residues do not affect the expression of M2e [44], and the double substitution does not affect the immunogenic properties of M2e epitopes [1].

It is interesting to note that in the vast majority of successful M2e vaccine studies mouse models were used, while only a few studies were conducted on other animals (pigs, ferrets, monkeys, dogs), where protection against challenge, if studied, was modest at best. One possible explanation for such diverse results is that mice of various inbred (including knockout and knock-in) strains are readily available at relatively low cost, whereas larger animals are more expensive and genetically outbred, resulting in significant host response variability. Even ferrets within commercial populations have distinct patterns of T-cell reactivity as a result of the heterogeneity at the MHC locus [21]. Furthermore, primary structure, cellular specificity and binding properties of Fc receptors can vary considerably among different mammalian species [54]. For example, a recent study found that pig Fc receptors do not bind human IgG1, which limits the use of this animal model to study human broadly protective monoclonal antibodies [85].

In summary, there are many ways to increase the immunogenicity of M2e. The most significant findings from preclinical studies of these universal vaccine prototypes are shown in Table 2. However, their further use in clinical practice is limited by the lack of knowledge about the safety of the vectors for humans. Currently, the search for new carriers continues, in order to increase the immunogenicity of M2e, while new immunization strategies are also being sought, such as priming with live attenuated influenza virus, and boosting with M2e VLP [73].

**Table 2:** Some examples of M2e-based vaccines

Carrier	Animals	Immunization route*	Main results	Reference
Hepatitis B virus core protein	Mice	IP^+^ or IN	3 immunizations at 3-week intervals with 5, 10, or 50 μg led to the formation of M2e-antibodies in mice. Groups immunized with 10 μg IP and IN in subsequent vaccination studies were protected after challenge with 5 LD_50_ of A/PR/8/34 (H1N1) and A/Victoria/3/75 (H3N2). The significant role of M2e antibodies in passive transfer experiments was shown.	[88]
	Mice	IM^+^	3 immunizations at 2-week intervals with 50 μg induced high levels of M2e-antibodies. Challenge with 5 LD_50_ of different heterologous viruses showed high degree of protection.	[122]
Modified form of the leucine zipper of the yeast transcription factor GCN4	Mice	IP^+^ or IN^+^	3 immunizations with 10 μg doses led to the formation of specific IgG1 and IgG2a M2e antibodies. The tetrameric M2e-tGCN4 vaccine induced M2e-specific IgG antibody that recognized natural M2 ectodomain. Immunized mice were fully protected against challenge with 4 LD_50_ X47.	[17]
Prime with M2-DNA and boost with recombinant adenovirus expressing M2	Mice	IM	2 immunizations (50 μg each) led to the enhanced antibody response. Challenge with LD_50_ of A/PR/8/34 (H1N1) and 10 LD_50_ of A/FM/1/47-MA (H1N1) and LD_50_ A/Thailand/SP-83/2004 (H5N1) showed the significant cross-protection of the vaccine. The important protective role of CD4 + and CD8 + cells was also shown.	[119]
T7 bacteriophage nanoparticles	Mice	SC^+^	3 immunizations (10^9^ PFU each) led to the formation of IgG1 and IgG2a M2e-antibodies, as well as a T-cell response. Challenge with 4 LD_50_ of A/PR/8/34 (H1N1) and X47 showed a high degree of protection.	[38]
Rotavirus fragment NSP4_98–135_	Mice	SC^+^	3 immunizations (10 μg each) with a chimeric protein resulted in the formation of an increased level of antibodies compared with immunization with M2e peptides. The formation of IgG1 M2e antibodies and to a lesser extent IgG2a was induced. Challenge using 3 LD_50_ of A/PR/8/34 (H1N1) or A/equine/London/72 (H7N7) did not reveal significant differences between the chimeric and peptide vaccine, however lung virus titers 3 d.p.i. were significantly lower in the M2e-NSP4 group.	[3]
Keyhole limpet hemocyanin (KLH) or *Neisseria meningitides* outer membrane protein complex (OMPC)	Mice	SC or IM^+^	3 immunizations (20 μg each) at 4-week intervals led to the formation of high levels of antibodies with cross-reactivity. Challenge with LD_90_ of A/Hong Kong/68xPR8 reassortant resulted in complete survival and lower weight loss in vaccinated mice compared with controls.	[28]
	Ferrets	IM^+^	3 immunizations (100 μg each) at 4-week intervals showed that the OMPC-based vaccine was more immunogenic than the KHL-based vaccine. Challenge with 100 TCID_50_ A/PR/8/34 (H1N1) revealed significantly lower replication of the challenge virus in the nasal turbinates and lungs.	
	Rhesus monkeys	IM^+^	3 immunizations (10 μg each) during 25 weeks (immunization on 0, 8, and 25 weeks) with an OMPC-based vaccine led to the formation of an increased level of M2e antibodies. Sera were examined after challenge with A/Hong Kong/68xPR8 after passive transfer immunization of mice, and protective efficacy was shown.	
*Brucella abortus* lumazine synthase protein (BLS)	Mice	SC, SC^+^, IM, IM^+^, IN, IN^+^	3 immunizations (10 μg each) using various routes at 3-week intervals led to the formation of IgG1 and IgG2a M2e-antibodies in different ratios. SC+ immunization produced the highest level of antibodies and was chosen for further study. Challenge with 5 LD_50_ of A/PR/8/34 (H1N1) showed the protective efficacy of the vaccine.	[2]
Malva mosaic virus nanoparticles	Mice	SC^+^	2 immunizations (20 μg each) at a 2-week interval led to the formation of IgG1 and IgG2a M2e-antibodies, whereas immunization with M2e peptides was not immunogenic. Significantly lower replication of the challenge virus in nasal turbinates and lungs was shown after challenge with A/WSN/1933 (H1N1).	[70]
	Dogs	IM^+^	3 immunizations (80 μg each) at 3-week intervals led to the formation of cross-reactive M2e-antibodies and revealed the need for adjuvant. Challenge was not performed.	
H1N1 HA DNA	Mice	IM^+^	2 immunizations (0.2 μg each) at a 3-week interval led to the formation of cross-reactive M2e-antibodies. High protection of immunized mice was shown against challenge with 5 LD_50_ of A/Aquatic Bird/Korea/W81/2005. In addition, HA-specific CD8+ and M2e-specific T cell responses were elicited	[92]
*Salmonella typhimurium* flagellin	Mice	SC	3 immunizations (6 μg each) at 3-week intervals led to the formation of a high level of M2e-antibodies. Full protection of vaccinated mice was shown against challenge with 10 LD_50_ of A/Aichi/2/68 (H3N2).	[113]
	Mice	SC or IN	2 immunizations (3 μg each) at a 2-week interval led to the formation of a higher rate of M2e-antibodies than immunization with M2e-peptides. There was no decline in the following 10 months. High protection of immunized mice was shown against challenge with LD_90_ of A/PR/8/34 (H1N1).	[46]
	Rabbits	IM	2 immunizations (15 μg each) at a 3-week interval led to the formation of M2e-antibodies.	
Multiple antigenic peptide	Mice	SC^+^	Single immunization led to the formation of high levels of M2e antibodies, which insignificantly declined in the following 6 months. 2 immunizations led to significant clearance of virus 3 days after challenge with 10 LD_50_ A/Beijing/501/09 and protected against weight loss.	[141]
DNA expressing fusion M2e-NP protein	Pigs	SC^+^	3 immunizations (200 μg each) at 3-week intervals did not protect animals after challenge with 10^8^ TCID_50_ of A/Sw/Best/96 (H1N1) but led to more serious signs of disease compared with the control group.	[41]
Lipopeptides	Mice	SC	2 immunizations (20 nmol each) at a 2-week interval with shortened form of M2e (a.a. 2–16) led to the same level of M2e antibody production as immunization with full-length M2e (a.a. 2–24), and led to lower viral titers in lungs and nasal turbinates after challenge with 10^4.5^ PFU of A/Memphis/1/71xA/Bellamy/42 (H3N1) virus.	[94]
Keyhole limpet haemocyanin (KLH with full length M2e (M2e-KLH) and M2e_2–10_ (SP1-KHL)	Mice	IP^+^	3 immunizations at 3-week intervals led to the formation of M2e-antibodies for both vaccines. Vaccinated groups were more protected than the control group against challenge with 4 LD_50_ of A/PR/8/34 (H1N1). M2e-KLH was more immunogenic and protective than SP1-KHL.	[18]
	Rabbits	n/m	3 immunizations at 3-week intervals showed greater immunogenicity of SP1-KHL. Serum from immunized rabbits provided protection in a mice passive transfer study against challenge with 4 LD_50_ of A/PR/8/34 (H1N1). SP1-KHL was also more immunogenic in outbred New Zealand white rabbits than in inbred BALB/c mice.	
VLP	Mice	IM	2 immunizations at a 4-week interval led to the formation of M2e-antibodies, and protected mice against challenge with 4 LD_50_ of A/Philippines/2/82(H3N2) 4 weeks and 8 months after boost.	[58]
M13 phage	SPF chickens	IM^+^ (1st), IM (2nd)	2 immunizations with the hybrid phage expressing shortened form of M2e (a.a. 2–9) at a dose of 1 × 10^10^ phage/200 μL produced specific antibodies against M2e (2–9) in broiler chickens.	[80]
CTA1-DD	Mice	IN	2 immunizations at 3-week intervals induced strong M2e-specific serum antibody response and stimulated significant anti-M2e IgA antibody titers in bronchial lavage. Vaccination provided strong protective immunity against challenge with 4 LD_50_ of X47 virus.	[25]
8C6 and 1B12 antibodies (recognize M2e_6–13_)	Mice	IP (passive transfer)	Passive transfer with 8C6 and 1B12 led to the formation of a high level of M2e antibodies and 75% protection of vaccinated mice against challenge with 5 LD_50_ of A/PR/8/34 (H1N1), compared with 0% protection in control group.	[78]
M2e-specific IgG2a MAb65	Mice	IP (passive transfer)	Passive immunization reduced transmission of A/Udorn/72 (H3N2) and A/Hong Kong/68 (H3N2) challenge viruses and led to lower viral titer in lungs and nasal turbinates.	[60]

* IP, intraperitoneal: IN, intranasal; SC, subcutaneous; IM, intramuscular; n/m, not mentioned; ^+^, with adjuvant

## Clinical studies of M2e-based influenza vaccines

M2e-based vaccines are not available commercially, but some are currently undergoing clinical trials (Table 3). For example, the ACAM-FLU-A vaccine, an M2e-HBc fusion protein, based on an idea by Neirynck [88], has already passed the first phase of double-blind placebo-controlled clinical trial (NCT00819013, Sanofi). Intramuscular administration of ACAM-FLU-A promoted the formation of anti-M2e antibodies in blood sera in 90% of cases and was well tolerated when given alone or with aluminum hydroxide or QS-21 Stimulon adjuvant (www.biocentury.com/bc-week-review/clinical-results/2008-01-07/acam-flu-phase-i-data). However, there has been no further progress with this vaccine since then, probably because of the rapid decline in M2e-specific antibody over time [*clinicaltrials.gov* NCT00819013]. A similar recombinant protein HBc-based prototype universal influenza vaccine, “Uniflu”, is currently being evaluated in a single-site, randomized, double-blind, placebo-controlled study in Saint Petersburg, Russia [*clinicaltrials.gov* NCT03789539]. The vaccine contains four copies of human M2e fused within the immunodominant loop of the HBc antigen [122]. This study involves 54 healthy adult subjects 18–60 years, and the vaccine is administered intramuscularly in two different doses (20 and 40 μg) twice at a 3-week interval. The results of the trial are anticipated in the near future.

**Table 3:** Clinical trials of M2-based vaccines

Company (country)	Phase	Year	Clinicaltrials.gov identifier	Available results
VaxInnate (USA)	I	2007–2008	NCT00603811	No
Sanofi (France)	I	2007–2009	NCT00819013	Yes
VaxInnate (USA)	I	2009	NCT00921206	Yes [123]
VaxInnate (USA)	II	2009–2011	NCT00921947	Yes
VaxInnate (USA)	I/ II	2009–2010	NCT00921973	Yes [117]
Imutex Limited (United Kingdom)	I	2010	NCT01181336	No
GeneOne Life Science (Republic of Korea)	I	2010–2012	NCT01184976	No
Theraclone Sciences (USA)	I	2012	NCT01390025	Yes [99]
	II	2012	NCT01719874	
VA Pharma LLC (Russian Federation)	I	2018	NCT03789539	No

Another influenza vaccine based on the use of flagellin to increase the immunogenicity of M2e [46] was shown to be safe in a phase I clinical trial after intramuscular administration in doses of 0.3 and 1.0 μg. However, administration in doses of 3.0 μg or 10.0 μg was accompanied by undesirable symptoms (fever, diarrhea, fatigue, headache and muscle pain), although this vaccine demonstrated high immunogenicity (NCT00921206, VaxInnate) [123]. The phase II trial of this vaccine ended in 2011 (NCT00921947, VaxInnate), but the test results are not yet available.

One more vaccine, containing M2 peptides and various conservative CTL epitopes [114], also passed the first phase of clinical trials (NCT01181336, Imutex Limited). However, this vaccine stimulates cellular immunity and is HLA-specific, which may be a reason for its narrow focus. Also, the cellular immune response is slower than the humoral response, and in the event of a pandemic, it would not effectively prevent infection [59].

Clinical trials to study the effect of the presence of additional M2e antigens in the trivalent vaccine have shown that, in the case of a weak response to the main vaccine, the M2e component can significantly enhance the immune response [117]. Thus, additional M2e epitopes will be able to have a “safety net” function when such vaccines are introduced into wide practice.

The TCN-032 antibody passed phase I of clinical trials. Volunteers were given a single dose of TCN-032 intravenously (1, 3, 10, 20, or 40 mg/kg of body weight), and no severe events related to the study drug were observed. A phase II clinical trial showed that non-neutralizing antibody TCN-032 has a therapeutic effect, as parenteral administration provided immediate immunity and therapeutic benefit in influenza A infection [99].

Overall, many prototype universal influenza vaccines contain an increased number of M2e epitopes. The data suggest that there is potential to use such vaccines, but that they need to be improved, in order to overcome the existing shortcomings.

## Conclusions

Vaccination is the safest and most effective way to prevent the spread of influenza. However, seasonal vaccination is ineffective against pandemic influenza viruses, as it is often antigenically different from the pandemic virus, and it takes an average of six months to prepare a new vaccine candidate for deployment. The development of a universal influenza vaccine, which would have a long and broad spectrum of action, is an urgent issue of practical public health.

Currently, new universal influenza vaccines are being developed using various strategies, but none of these vaccines has been licensed so far. The development of a new universal influenza vaccine will both avoid the need for annual updates to the composition of seasonal vaccines, and reduce the risk of public health disaster in the event of a new pandemic. However, it is not yet clear whether such vaccines will induce long-lasting protection. It is also unknown how the effects of influenza viruses and previous vaccinations will affect the performance of new vaccines [91].

Ideally, a universal influenza vaccine should induce both a humoral (antibodies) and a T-cell immune response to conservative epitopes of the influenza virus. M2e and HA stalk domain are the most widely used viral targets for a universal influenza vaccine design due to their conserved nature and the proven ability of their specific antibody to protect against heterologous viral infections. Many vaccination strategies have been explored to elicit potent antibody responses against these naturally weak immunogens. The vaccine-induced anti-HA stalk antibodies target either group 1 or group 2 HAs, with limited cross-reactivity between the two HA groups, suggesting that a cHA-based universal influenza vaccine has to include three components: a group 1 HA, a group 2 HA, and an influenza B stalk-based antigen [64, 82]. In contrast, a properly designed M2e-based vaccine should cover all influenza A virus subtypes circulating in both human and animal reservoirs. Since it is not possible to predict which virus will cause the next pandemic, we recommend that four different M2e consensus sequences be included in the new M2e-based vaccines to maximize cross-reactivity of the M2e-targeted antibody against all circulating influenza A viruses (Fig. 1). Licensing of a new M2e-based vaccine is challenging due to the lack of clearly defined correlates of protections of such vaccines for humans. In addition, the M2e-specific protection is of lower potency compared to HA-targeted neutralizing immunity, therefore the problem of proving non-inferiority over existing influenza vaccines remains to be solved. Nevertheless, the conserved M2 ectodomains can be used as a supplement to overcome strain specificity and improve long-term cross-protection of currently licensed seasonal influenza vaccines [57, 87].

It is worth mentioning that it is possible that a new antigenic drift, different from that observed in wild strains, will start to act on conservative epitopes, which will make it necessary to periodically update the composition of any universal vaccine. However, such an update will be required less frequently than is currently the case for seasonal vaccines, as there is evidence that changes affecting the conserved M2e region also require changes in other influenza A viral proteins because of the various functions of M2 in the virus life cycle.

## Data Availability

The datasets used and/or analysed during the current study are available from the corresponding author on reasonable request.

## Abbreviations

A.a.: Amino acid
ADCC: Antibody-dependent cellular cytotoxicity
Bac-AM2: Baculovirus containing M2 from influenza virus A/Ann Arbor/6/60
BLS: *Brucella abortus* lumazine synthase protein
CTL: Cytotoxic T lymphocyte
ESCRT: Endosomal sorting complexes required for transport
FcR: Fc receptor
HA: Hemagglutinin
HBc: Hepatitis B core protein
IM: Intramuscular immunization
IN: Intranasal immunization
IP: Intraperitoneal immunization
KLH: Keyhole limpet hemocyanin
LLC: Limited Liability Company
M2e: Extracellular domain of the M2 protein
M2e5x: Five M2e tandem repeats
M2HBc: Hepatitis B core protein combined with the M2e peptide
M2SR: M2-deficient single replication virus
NA: Neuraminidase
NK: Natural killer
NP: Nucleoprotein
OMPC: Outer membrane protein complex
SC: Subcutaneous immunization
Sf9: *Spodoptera frugipedra*
VLP: Virus-like particles
